# The role and mechanism of butyrate in the prevention and treatment of diabetic kidney disease

**DOI:** 10.3389/fmicb.2022.961536

**Published:** 2022-08-09

**Authors:** Xi Cheng, Tingting Zhou, Yanqiu He, Yumei Xie, Yong Xu, Wei Huang

**Affiliations:** ^1^Department of Endocrinology and Metabolism, Metabolic Vascular Diseases Key Laboratory of Sichuan Province, Affiliated Hospital of Southwest Medical University, Luzhou, China; ^2^Sichuan Clinical Research Center for Nephropathy, Luzhou, China; ^3^Cardiovascular and Metabolic Diseases Key Laboratory of Luzhou, Luzhou, China

**Keywords:** butyrate, diabetic kidney disease, immune, inflammation, epigenetics

## Abstract

Diabetic kidney disease (DKD) remains the leading cause of the end-stage renal disease and is a major burden on the healthcare system. The current understanding of the mechanisms responsible for the progression of DKD recognizes the involvement of oxidative stress, low-grade inflammation, and fibrosis. Several circulating metabolites that are the end products of the fermentation process, released by the gut microbiota, are known to be associated with systemic immune-inflammatory responses and kidney injury. This phenomenon has been recognized as the “gut–kidney axis.” Butyrate is produced predominantly by gut microbiota fermentation of dietary fiber and undigested carbohydrates. In addition to its important role as a fuel for colonic epithelial cells, butyrate has been demonstrated to ameliorate obesity, diabetes, and kidney diseases *via* G-protein coupled receptors (GPCRs). It also acts as an epigenetic regulator by inhibiting histone deacetylase (HDAC), up-regulation of miRNAs, or induction of the histone butyrylation and autophagy processes. This review aims to outline the existing literature on the treatment of DKD by butyrate in animal models and cell culture experiments, and to explore the protective effects of butyrate on DKD and the underlying molecular mechanism.

## Introduction

Diabetic kidney disease (DKD) is a serious microvascular complication of diabetes mellitus and is also the leading cause of the end-stage renal disease (Marshall, 2016). Based on data from International Diabetes Federation, over 40% of patients with diabetes will develop DKD. This causes an increase in health care costs, cardiovascular events risks, and mortality. The etiology and pathogenesis of DKD are complex and have not been completely understood. It mainly includes hemodynamic changes, oxidative stress, insulin resistance, and the release of pro-inflammatory cytokines. All these events lead to glomerulosclerosis, tubular atrophy, fibrosis, and irreversible renal injury (Kawanami et al., 2016). Thus, the prevention and effective treatment of DKD is crucial and the most challenging aspect of clinical studies.

Short-chain fatty acids (SCFAs) are the main metabolic products by the bacterial fermentation of macro-fibrous material that escapes digestion and enters the colon. 90 to 95% of SCFAs in the colon are composed of acetate, propionate, and butyrate (Cummings et al., 1987). Among these SCFAs, butyrate is of particular concern due to its positive influence on cell energy metabolism and intestinal environmental stability (Guilloteau et al., 2010). It also relieves oxidative stress, inflammation, and fibrosis in diabetes and kidney diseases *via* G-protein coupled receptors (GPCRs) or serves as an epigenetic regulator by inhibiting histone deacetylase (HDAC), up-regulation of miRNAs, or induction of the histone butyrylation, a novel histone post-translational modification (Lin et al., 2015; Lu et al., 2016; Xu et al., 2018; Gonzalez et al., 2019; Sanna et al., 2019; Elgamal et al., 2020; Noureldein et al., 2020; Rodríguez-Carlos et al., 2020). Studies have already reported the therapeutic effects of butyrate or sodium butyrate, demonstrating that butyrate metabolic pathway could be a new therapeutic target for DKD (Du et al., 2020a,b; Huang et al., 2020; Li et al., 2020). This article aims to provide an overview of the role and the latent mechanism of butyrate action on DKD.

## Overview of butyrate and butyrate-mediated responses

### Origin, production, transport, and distribution of butyrate

**Figure 1:**
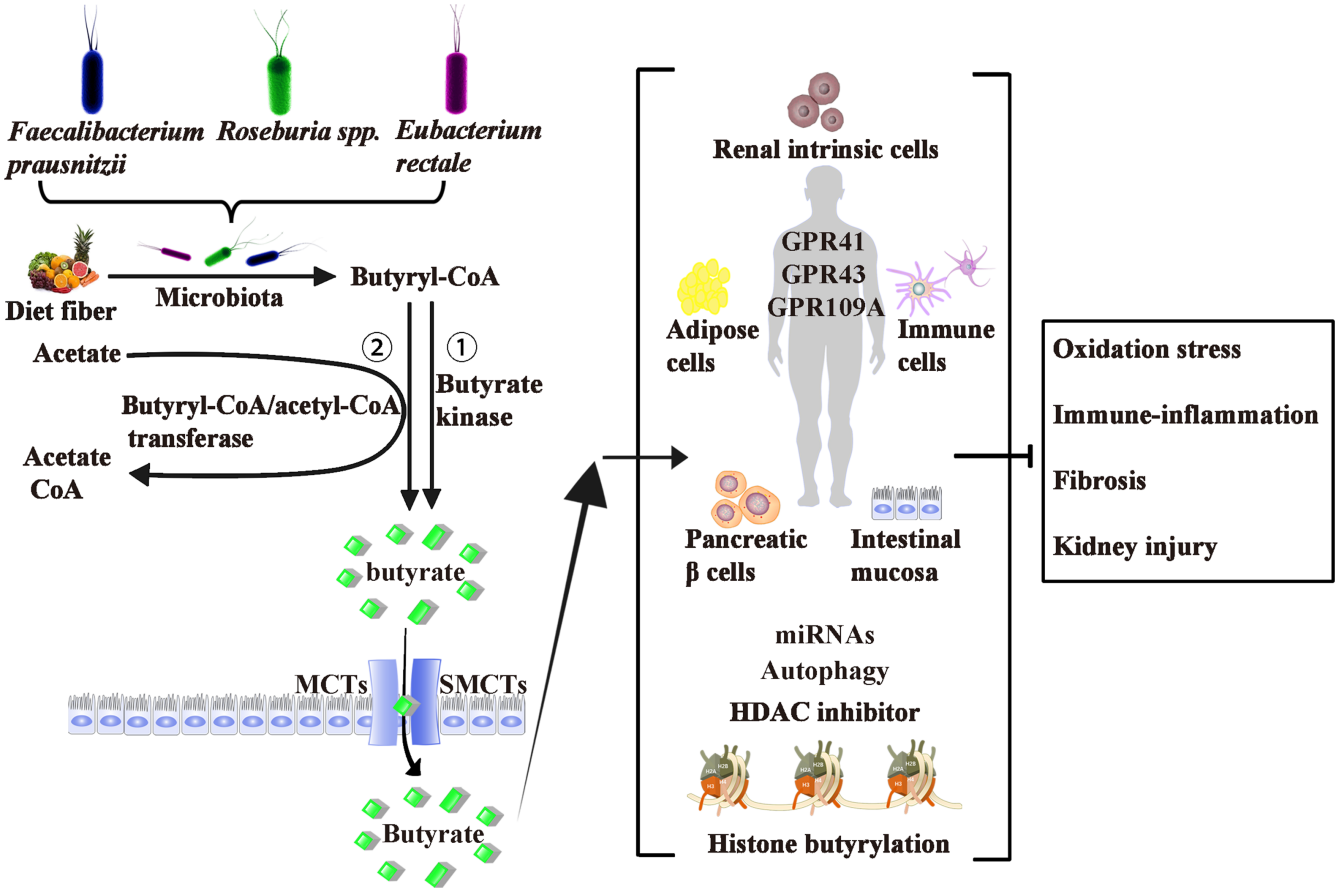
Origin, Production, Transport, Effects, and Mechanism of Butyrate. Butyrate is produced from dietary fiber by bacterial fermentation through two metabolic pathways: (1) butyryl-CoA is transformed to butyrate *via* butyrate kinase and (2) the CoA moiety of butyryl-CoA is transferred to butyrate and acetyl-CoA *via* butyryl-CoA: acetate CoA-transferase. The two most important butyrate-producing bacteria are *Faecalibacterium prausnitzii* and *Eubacterium rectale/Roseburia* spp. Butyrate is absorbed by colonic epithelial cells as energy sources mainly through MCTs and SMCTs. About three of the de-orphanized GPCRs (GPR41, GPR43, and GPR109A) have been identified as butyrate receptors in the human intestinal mucosa, renal intrinsic cells, immune cells, pancreatic β cells, and adipose tissues. Butyrate act as epigenetic regulators by the inhibition of HDAC, the upregulation of miRNAs, or induction of the histone butyrylation and autophagy. Although controversial, most studies believe that exogenous or endogenous butyrate improves inhibits oxidative stress, and ameliorates diabetic inflammation. GPCRs, G-protein coupled receptors; MCTs, monocarboxylate transporters; SMCTs, sodium-coupled monocarboxylate transporters; CoA, coenzyme A; HDAC, histone deacetylase; miRNAs, microRNAs.

As shown in Figure 1, butyrate is generally thought to be generated by bacterial fermentation of dietary fiber that enters the colon after digestion in the upper gastrointestinal tract (Bergman, 1990; Simpson and Campbell, 2015). The bacteria involved in butyrate production are widespread, the two most important clusters being *Faecalibacterium prausnitzii* in the *Clostridium leptum* cluster and *Eubacterium rectale/Roseburia* spp. in the *Clostridium coccoides* cluster of *Firmicutes* (Mokhtari et al., 2017). In a normal human feces sample, each typically accounts for 5–10% of the total microbial load that may be detected (Haenen et al., 2013). Some of these butyrate-producing bacteria are also scattered in clusters IX, XV, XVI, and XVII (Haenen et al., 2013).

Butyrate is produced *via* two metabolic pathways: firstly, butyryl-coenzyme A (CoA) is phosphorylated to form butyryl-phosphate, which is then converted to butyrate *via* butyrate kinase (Louis and Flint, 2017). Secondly, the CoA moiety of butyryl-CoA is transferred to butyrate and acetyl-CoA *via* butyryl-CoA: acetate CoA-transferase (Trachsel et al., 2016). The butyrate’s absorption mechanisms through the apical membrane of colon cells include monocarboxylate transporters and sodium-coupled monocarboxylate transporters (Kumar et al., 2015; Counillon et al., 2016). These transporters are diffusely expressed from the central nervous system to peripheral tissues, including liver, fat, heart, and kidney tissues.

### Butyrate ameliorates metabolic disorder and kidney injury

Studies have demonstrated a protective effect of butyrate by improving body weight, blood glucose, lipid distribution, and insulin sensitivity in animal models of obesity and diabetes, which can be postulated as new therapeutic strategy to counteract obesity and insulin resistance (Gao et al., 2009; Khan and Jena, 2016; Mollica et al., 2017; Hu et al., 2018). The butyrate-mediated secretion of glucagon-like peptide-1 may mediate the reduction of insulin sensitivity and diabetes (Bridgeman et al., 2021). Glucagon-like peptide-1 has been shown to reduce hepatic gluconeogenesis and stimulate insulin secretion (Jin and Weng, 2016). Increased levels of butyrate-induced glucose transporter 4 in adipose tissues is also a crucial factor hypothesized to be responsible for the improvement in levels of glucose metabolites (Si et al., 2018). The current studies suggest that butyrate improves acute-chronic kidney injury that has been induced by ischemia–reperfusion (Zheng et al., 2019; Sun et al., 2022), contrast agent (Machado et al., 2012), gentamicin (Sun et al., 2013), doxorubicin (Felizardo et al., 2019), hypertension (Wu et al., 2021), and lipopolysaccharide (LPS; Huang et al., 2017; Dou et al., 2022). It acts on kidney inflammation, immunity, fibrosis, and energy metabolism and has a protective effect on the kidneys. Thus, the role of butyrate in kidney disease is an intriguing area of research.

### Butyrate ameliorates kidney injury through the “gut-kidney axis”

A growing number of studies have suggested that kidney inflammation is related to bacterial load and endotoxins produced by gastrointestinal dysbiosis, which increases gut permeability and contributes to infection-related acute renal failure and chronic accelerated development of kidney disease (Li et al., 2019; Yang et al., 2019; Cai et al., 2020; Snelson et al., 2021). Several studies have observed significant differences in gut microbiota richness and populations between DKD patients and healthy controls (Jiang et al., 2016; Castillo-Rodriguez et al., 2018; Hu et al., 2020). This connection between the gut and kidney has been termed the “gut-kidney axis” (Leonel and Alvarez-Leite, 2012; Evenepoel et al., 2017).

A study by Terpstra ML (Terpstra et al., 2019) did not find significant differences in the number or capacity of the three most abundant butyrate-producing bacteria (*F. prausnitzii*, *E. rectale,* and *Roseburia spp*) between end-stage renal disease patients and healthy kidney donors. However, butyrate has been shown to make a positive contribution to kidney disease in humans. A recent clinical study by Wang et al. (2019) indicated reduced SCFA levels in patients with chronic kidney disease (CKD), suggesting that butyrate supplementation may delay the progression of CKD. Of further interest is a recent study that showed that quantitative reduction in SCFAs, especially butyrate, contributed to the progression of CKD (Wang et al., 2019). In a study by Cai et al. (2022), decreased serum butyrate levels were noted in the DKD group. Also, dysbiosis was evident in the gut microbiota of DKD patients, particularly associated with the lower abundance of SCFAs-producing bacteria belonging to the *Ruminococcaceae, Lachnospiraceae, and Butyricicoccus* groups. Li et al. (2022) also observed that the butyrate in serum was inversely connected with DKD. Given the substantial association between gut microbiota and butyrate, randomized controlled studies are necessary to examine whether alteration in the gut microbiota prevents DKD. Despite the controversy, the relationship between butyrate levels and renal injury has been proposed, indicating that butyrate may be a prospective target for the treatment of DKD. New mechanisms are being uncovered demanding the investigation of the role of endogenous butyrate on the “gut-kidney axis.”

## The molecular mechanism of butyrate-mediated renal protection

Butyrate modulates the host’s biological responses mainly through the following mechanisms: (1) Butyrate directly inhibits HDAC (enzymes that remove acetyl groups from histone tails and regulate gene expression; Bose et al., 2014; Bridgeman et al., 2021). (2) Butyrate is involved in signalling through metabolite-sensing GPCRs. Three butyrate receptors have been identified, namely GPR41, GPR43, and GPR109A, out of the ten trophic receptor GPCRs (Miyamoto et al., 2016). (3) Butyrate may increase autophagy by switching on the AMP-activated protein kinase (AMPK)/mammalian target of rapamycin (mTOR) pathway (Cai et al., 2022). (4) The epigenetic mechanisms such as butyrate-mediated histone butyrylation (Goudarzi et al., 2016) and microRNAs (miRNAs; Yang et al., 2021) also modulate biological processes and are being extensively scrutinized. The effects and mechanisms of butyrate metabolism relevant to the “gut-kidney axis” and DKD will be discussed further in this part of the review.

### GPR41 and GPR43

GPR41 and GPR43 have been identified as receptors for SCFAs, hence named free fatty acid receptors 3 and free fatty acid receptors 2, respectively (Brown et al., 2003). A previous study (Huang et al., 2020) by our research group evaluated the effects of three major SCFAs (acetate, propionate, and butyrate) on a high-fat diet and streptozotocin (STZ)-induced type 2 diabetes and DKD mouse models. The role and mechanism of butyrate in high glucose (HG)-induced mouse glomerular mesangial cells were explored to decipher new therapeutic strategies and molecular targets for DKD. We demonstrated that exogenous SCFAs, especially butyrate, partially ameliorated type 2 diabetes-induced kidney injury through GPR43-mediated inhibition of oxidative stress and nuclear factor kappa B (NF-κB) signalling. Our study suggested that butyrate may be a potential therapeutic agent for the prevention and treatment of DKD (Huang et al., 2020).

Both GPR41 and GPR43 activate the heterotrimeric G protein and subsequently unite with β-arrestins (β-arrs), which regulate the desensitization, internalization, intracellular signal transduction, and recirculation of GPCRs, and are responsible for the inflammation signalling pathway (Lee et al., 2013; Eichel et al., 2018). Huang et al. (2020) showed that HG induced the expression of βarr-2, but not βarr-1, and HG reduced the interaction between βarr-2 and I-κBα. However, this effect was reversed by butyrate *via* GPR43, suggesting that GPR43-β-arrestin-2 signalling could be a prospective target for DKD treatment. Nevertheless, numerous questions regarding the functionality of butyrate remain unanswered and disputed. Future research on GPR41 and GPR43 will require more effective and selective tools.

### GPR109A

GPR109A was first identified as a niacin receptor activated by β-hydroxybutyrate and butyrate (Walters et al., 2009). As a ligand for GPR109A, butyrate reduces intestinal inflammation and promotes the integrity of the intestinal epithelial barrier, so activation of GPR109A is thought to have a protective effect (Macia et al., 2015; Feng et al., 2018). One study (Li et al., 2020) suggested that dietary fiber prevents DKD by regulating the intestinal flora, enriching SCFAs producing bacteria, and increasing SCFAs production, GPR43−/− and GPR109A−/− mice were sensitive to STZ-induced diabetes, indicating that GPR43 and GPR109A are indispensable for fiber and butyrate mediated protection against DKD. However, the mechanism of GPR109A in the pathogenesis and treatment of DKD needs further characterization.

### HDAC inhibitor

Acetate, propionate, and butyrate have all been reported as HDAC inhibitors, with butyrate being the most widely studied. Also, amongst all SCFAs, butyrate was mentioned to have the most potent inhibitory effect on HDAC activity *in vitro* and *in vivo*. The maximum inhibition effectiveness of about 80% was observed for HDAC1/2, while that of propionate was about 60% (Corfe, 2012). Several reported effects of butyrate have been attributed to epigenetic effects that regulate the expression of genes in activated T cells, including NF-κB, myoblast antigens, p53, and nuclear factors, by inhibiting HDAC, increasing histone acetylation, and reducing histone densification (Wang and Friedman, 2000; Cruz-Bravo et al., 2014).

The mechanism of inhibition of HDAC activity by butyrate remains unclear. However, its benefits may be related to its anti-fibrosis, anti-inflammatory, and immunosuppressive effects in polycystic kidney disease (Vinolo et al., 2011; Manson et al., 2014; Khan et al., 2015). Oxidative stress contributes to the pathogenesis of DKD (Zhang et al., 2012; Keshari et al., 2015). Du et al. (2020a) found that sodium butyrate acts as an antioxidant and suppresses the HG-induced apoptosis in normal rat kidney tubular epithelial cells by inhibition of HDAC2. Sodium butyrate is also an activator of nuclear factor erythroid 2-related factor 2 (Nrf2, Yaku et al., 2012; Wu et al., 2018). Dong et al. (2017) first noted that Nrf2 was an important aspect of sodium butyrate’s mediated protection against DKD. Sodium butyrate may inhibit HDAC activity, facilitate the expression of the Nrf2 gene, which might enter the nucleus, and upregulate downstream targets-HO1 (heme oxygenase 1) and NQO1 (NAD (P) H dehydrogenase quinone 1). It also inhibits oxidative stress and inflammation in DKD. In contrast, in the absence of the Nrf2 gene, the role of sodium butyrate is eliminated. Although sodium butyrate seems to play a significant role *via* the activation of Nrf2, further studies are necessary before sodium butyrate can be suggested for use in humans.

The coagulation protease-activated protein C studies showed cytoprotective effects *in vitro* and *in vivo* disease models, including DKD (Madhusudhan et al., 2020). Activated protein C reduced glucose-induced hypomethylation and hyperacetylation of the p66^Shc^ promoter in podocytes, sodium butyrate eliminated the protective effect of protease-activated protein C (Bock et al., 2013). Therefore, the role of butyrate as an HDAC modulator in the DKD is still controversial, and it is necessary to understand the mode of action of butyrate in intestinal physiology and lipid metabolism. The research will contribute to the consideration of butyrate as an HDAC inhibitor in preventing and treating DKD kidney injury.

### Regulation of miRNAs

Recently, long non-coding RNAs (lncRNAs; Vinolo et al., 2011) and miRNAs (Du et al., 2020b) have been paid elaborate attention to decipher the molecular mechanisms of butyrate-mediated renal protection. miRNAs are small non-coding RNAs that regulate gene expression through the 3′ untranslated region of messenger RNAs (mRNAs). In DKD, extensive alterations in the expression of miRNAs such as miR-184 (Zanchi et al., 2017), miR-192 (Kato et al., 2013), and miR-21 (Kölling et al., 2017) was noted. Butyrate has been reported to influence miRNAs expression in oncogenic signalling pathways such as miR-92a, miR-22, miR-3,935, miR-574-3p, and miR-106B (Hu et al., 2015; Pant et al., 2017; Xiao et al., 2018). Du et al. (2020b) demonstrated that miR-7a-5p was prominently reduced in the HG-induced SV40-MES-13 cells and the kidneys of db/db mouse, while butyrate induced the upregulation of Mir-7a-5P. Later, the Mir-7a-5p inhibitors were administered to block the antifibrotic effects of butyrate.

Yang et al. (2021) also confirmed that sodium butyrate improved renal dysfunction in DKD model mice induced by db/db mice. Further RNA-seq results (Yang et al., 2021) showed that certain lncRNAs and mRNAs in the DKD+ sodium butyrate group had reverse changes compared with the DKD groups, and subsequent bioinformatics analysis also suggested that these changes might affect nephritis. These cumulative results indicated that sodium butyrate could protect DKD by altering the expression of lncRNA in mouse kidneys.

### Autophagy

Autophagy is a highly conserved process and maintains cellular homeostasis (Liu et al., 2017). The expression of autophagy-related protein 7 in renal tubular epithelial cells was increased by SCFAs, suggesting that SCFAs regulate autophagy in acute kidney injury (Andrade-Oliveira et al., 2015). In colorectal and bladder cells, sodium butyrate also stimulated autophagy and inhibited tumor growth (Zhang et al., 2016; Luo et al., 2019; Wang et al., 2020). These results suggest a correlation between SCFAs and DKD *via* autophagy signaling.

AMPK is activated and downregulates the mTOR pathway under hunger or increased energy needs, which may induce autophagy to maintain homeostasis (Alers et al., 2012). AMPK phosphorylation was activated by butyrate in CKD rats, reducing renal injury (Gonzalez et al., 2019). Also, sodium butyrate causes autophagy-mediated cell death and reactivates the tumor suppressor gene DIRAS1 in the UOK146 renal cell carcinoma cell line (Verma et al., 2018). By increasing intestinal barrier function and activating the free fatty acid receptor 2-mediated PI3K/AKT/mTOR pathway, butyrate protects against DKD-induced muscle atrophy (Tang et al., 2022). Cai et al. (2022) first revealed that sodium butyrate boosted the phosphorylation of AMPK (Thr172), inhibited the phosphorylation of mTOR (Ser2448), and initiated autophagy in DKD rats. However, extensive research is needed to evaluate the mechanisms and interactions involved in gene knockout or overexpression *in vitro* and *in vivo.*

### Histone butyrylation

We previously described that butyrate improves DKD by inhibiting HDAC (Khan and Jena, 2014; Dong et al., 2017; Du et al., 2020a). In the recent years, histone butyrylation (Chen et al., 2007), crotonylation (Tan et al., 2011), β-hydroxybutyrylation (Xie et al., 2016), and other novel histone post-translational modifications have been found. Pelletier N reported these mechanisms to cooperate or antagonize with histone acetylation and methylation, which plays a crucial role in gene expression regulation and cell fate decision (Pelletier et al., 2017). In 2007, Zhao Y’s team found that butyrate metabolite-butyryl-CoA, catalyzed by transcriptional coactivator P300 with acetyltransferase activity, can transfer the butyl group to the histone lysine side chain, and produce a novel modification-histone butyrylation (Chen et al., 2007). Yang et al. (2021) reported that butyrylation of histone H3K9 is negatively regulated by high-fat stress in mouse heart tissue. Also, Ishikawa-Kobayashi E and Gaikwad AB reported that the acetylation of H3K9 is involved in obesity and diabetic heart disease (Gaikwad et al., 2010; Ishikawa-Kobayashi et al., 2012). The β-hydroxybutyrylation of H3K9 up-regulated matrix metalloproteinase-2 and improved DKD (Luo et al., 2020). Nie et al. (2017) reported that the butyrylation of H3K18 in hepatocytes of high-fat induced obesity mice model significantly decreased, and the acetylation was up-regulated. The above studies thus suggest that the crosstalk between these modifications at the same lysine site may have synergistic or antagonistic effects on related gene expression. Since butyrate may induce butyrylation of histone, the role of this modification in the pathogenesis and prevention of DKD deserves further attention.

## Application of butyrate in prevention and treatment of DKD

In a limited number of investigations conducted to date, butyrate or sodium butyrate has been used therapeutically in DKD *in vivo* and *in vitro* studies. Research conducted on the application of butyrate in DKD is summarized in Table 1.

**Table 1:** Application of butyrate *in vivo* and *in vitro* model of DKD.

Type	Model species	Mode and dose	Results	References
	STZ-induced juvenile male SD rats	Sodium butyrate (500 mg/kg/day) by intraperitoneal injection	HDACs activity↓; BG↓; SCr↓, BUN↓; NOS↓, iNOS↓; a-SMA↓; collagen I↓; fibronectin↓; TGF-β1↓; NF-κB↓; apoptosis↓; DNA damage↓	Khan and Jena (2014)
	STZ-induced C57BL/6 mice and Nrf2−/− mice	Sodium butyrate diet (5 g/kg/day)	BG (−); UACR↓; mesangial matrix↓; TGF-β1↓; CTGF↓; PAI-1↓; HDAC activity↓; Nrf2↑	Dong et al. (2017)
	db/db mice	Sodium butyrate (1 g/kg/day) by oral gavage	BG (−); UACR↓; apoptosis↓ (BCL-2↑; Bax↓; caspase-3↓)	Du et al. (2020a)
	db/db mice	Sodium butyrate (1 g/kg/day) diet	BG (−); BW (−); mesangial matrix↓; UACR (↓); collagen IV↓; PAI-1↓; a-SMA↓; CTGF↓; P311↓; miR-7a-5p↑; TGF-β1↓	Du et al. (2020b)
*In*	STZ-induced C57BL/6 mice	Sodium butyrate 100 mg/ (kg·48 h) by intraperitoneal injection	BW (−); FINS (−); blood lipid spectrum (−); RBG↓; FBG↓; IR↓; UACR↓; SCr↓; BUN↓; cystatin C↓; NF-κB↓; renal fibrosis↓	Huang et al. (2020)
*vivo*	db/db mice	Sodium butyrate (5 g/kg/day) by intraperitoneal injection	BW↓; BG (−); UACR↓; glomerular and tubular injuries↓	Yang et al. (2021)
	STZ-induced C57BL/6 mice	Sodium butyrate (50 mM) dissolved and administered *ad libitum* in drinking water	BG (−); UACR↓; podocytes/glomerulus↓; collagen-PSR↓; macrophage (CD68+) ↓	Li et al. (2020)
	STZ-induced juvenile male SD rats	Sodium butyrate (8 g/l) dissolved and administered *ad libitum* in drinking water	H3 acetylation↑; nitrotyrosine accumulation↑; p66^Shc^↑, albuminuria↑	Bock et al. (2013)
	db/db mice	Sodium butyrate (1 g/kg/day) diet	Serum butyrate↑; SCr↓; BUN↓; and UACR↓; ZO-1↑; occludin↑	Tang et al. (2022)
	STZ-induced SD rats	Sodium butyrate (300 mg/kg) by oral gavage	Serum and fecal butyrate↑; BG↓; BUN↓; eGFR↓; fibronectin↓; collagen IV↓; LC3↑; LC3BII/I↑; autophagosomes↑; mTOR↓; AMPK↑	Cai et al. (2022)
	mouse kidney mesangial cell (SV40-MES 14 cells)	Sodium butyrate (5 mM)	lncRNA (+); mRNA (+)	Yang et al. (2021)
	Mouse glomerular mesangial cells (SV40-MES 13)	Sodium butyrate (5 mM)	Oxidative stress↓ (ROS↓, MDA↓, SOD↑); inflammation ↓ (ICAM-1↓, MCP-1↓, IL-1β↓)	Huang et al. (2017)
*In*	Mouse glomerular mesangial cells (SV40-MES 13)	Sodium butyrate (5 mM)	ROS↓; MDA↓; MCP-1↓; IL-1β↓; NF-κB activation↓	Huang et al. (2020)
*vitro*	Rat kidney tubular epithelial (NRK52E) cells	Sodium butyrate (0.1, 0.5, or 1.0 mM)	HDAC2↓; BCL-2↑; Bax↓; caspase-3↓; oxidative stress ↓ (ROS↓, SOD↑, LDH↓)	Dong et al. (2017)
	Mouse mesangial cells (SV40-MES-13)	Sodium butyrate (0.5 mM)	Collagen IV↓; PAI-1↓; a-SMA↓; CTGF↓; P311↓; miR-7a-5p↑; TGF-β1↓	Du et al. (2020b)
	Mouse kidney tubular epithelial cells and podocytes	Sodium butyrate (3.2 mM)	IL-6↓; fibronectin↓; TGF-β↓; TNF-α↓; MCP-1	Li et al. (2020)

(−) no effect; (+) have an effect; STZ, streptozotocin; HDAC, histone deacetylase; HG, high glucose; eNOS, endothelial nitric oxide synthase; iNOS, inducible nitric oxide synthase; a-SMA, alpha smooth muscle actin; UACR, the ratio of urinary albumin to creatinine; TGF-β, transforming growth factor-β; CTGF, connective tissue growth factor; PAI-1, plasminogen activator inhibitor-1; Nrf2, nuclear factor erythroid 2-related factor 2; FINS, fasting insulin; BG, blood glucose; FBG, fasting blood glucose; RBG, random blood glucose; BW, body weight; SCr, serum creatinine; BUN, blood urea nitrogen; eGFR, estimated glomerular filtration rate; lncRNA, long non-coding RNA; mRNA, messenger RNA; IL-1β, interleukin-1β; IL-6, interleukin-6; ROS, reactive oxygen species; MDA, Malondialdehyde; SOD, superoxide dismutase; LDH, lactate dehydrogenase; NF-κB, nuclear factor kappa B; ICAM-1, intercellular adhesion molecule 1; MCP-1, monocyte chemotactic protein 1; TNF-α, tumor necrosis factor-α; BCL-2, B-cell lymphoma-2; Bax, BCL-2-Acssocisted X; ZO-1, zona occludens-1; IR, insulin resistance; AMPK, AMP-activated protein kinase; mTOR, mammalian target of rapamycin; LC3, an autophagy marker.

### Butyrate ameliorates DKD as HDAC inhibitor

In a study by Khan S (Khan and Jena, 2014), sodium butyrate reduced plasma glucose, creatinine, urea, histological alterations, and descent of the HDACs activity. It also curbed the expression of endothelial nitric oxide synthase, inducible nitric oxide synthase, alpha-smooth muscle actin, collagen I, fibronectin, and transforming growth factor-β (TGF-β), and NF-κB in the kidneys of STZ-induced diabetic mice (Khan and Jena, 2014). However, Du Y’s study (Du et al., 2020a) showed that sodium butyrate as an HDAC2 inhibitor had no significance on the blood glucose levels of diabetic mice, though it significantly improved the ratio of urinary albumin to creatinine (UACR). Further, Dong et al. (2017) found that sodium butyrate did not alter blood glucose levels in STZ-induced diabetic C57BL/6 Nrf2 knockout and the WT mice, but significantly reduced UACR.

Surprisingly, Bock et al. (2013) reported that supplementing the drinking water with 8 g/l sodium butyrate eliminated the protective effect of activated protein C in diabetic mice. It further increased renal H3 acetylation, PAS-positive staining, nitrotyrosine accumulation, and urine albumin without affecting blood glucose levels or albuminuria in non-diabetic control animals. Chen et al. (2014) showed that in sodium butyrate-treated HBZY-1 cells (the rat mesangial cells), mRNA levels of monocyte chemotactic protein 1, intercellular adhesion molecule 1, and vascular cell adhesion molecule 1 significantly increased (1.7, 1.3 and 3.1 times). Apelin13, the most active member of the adipokine apelin group, inhibited sodium butyrate-induced inflammation and HDAC1 reduction in HBZY-1 cells by regulating histone acetylation (Chen et al., 2014). Therefore, a new mechanism was proposed to determine whether butyrate mediated inhibition of HDAC and contributed to the improvement of DKD. However, more studies are necessary to characterize and explain the controversial effects of endogenous butyrate.

### Butyrate mitigates DKD acts as GPCRs agonist

In addition to protecting against DKD by inhibiting the HDAC pathway, butyrate also acts as an agonist of GPCRs to ameliorate DKD. DKD is a chronic low-grade inflammatory disease, and SCFAs have proven to positively affect inflammation and kidney injury (Huang et al., 2017; Xu et al., 2018). Previous studies by Huang et al. (2017) showed that butyrate and GPR43 agonists reversed the HG and LPS-induced mesangial cell proliferation, reduced reactive oxygen species and malondialdehyde generation, and controlled the inflammatory cytokine release. Ensuing research (Huang et al., 2020) evaluated the effects of butyrate in mice fed with a high-fat diet, with STZ-induced type 2 diabetes, DKD, and on HG-induced mouse glomerular mesangial cells. The results showed that exogenous butyrate improved blood glucose and insulin resistance and avoided UACR, mesangial matrix accumulation, and renal fibrosis in mice. Butyrate is responsible for the aforementioned effects *via* GPR43-mediated suppression of oxidative stress and NF-κB signalling.

Snelson et al. (2020) first studied the effects of GPR109A deficiency on DKD through analysis of GPR109A signalling deficiency on the development of DKD renal injury by GPR109A gene deletion. The findings of the long-term study (24 weeks) suggested that GPR109A deficiency does not play a vital role in the occurrence and development of DKD and gastrointestinal homeostasis. On the contrary, Li et al. (2020) explored the protective effects of dietary fiber on experimental DKD in STZ-induced diabetic mice or GPR43/GPR109A knockout mice. The diabetic mice fed on a high-fat diet had lower rates of albuminuria, glomerular hypertrophy, podocyte injury, and interstitial fibrosis compared with diabetic controls. Intake of fiber beneficially increased faecal and systemic SCFA concentrations and decreased the expression of inflammatory factors, chemokines, and fibrosis-promoting proteins in diabetic kidneys.

Since butyrate is recognized by the GPR109A, GPR41, and GPR43 receptors (Miyamoto et al., 2016), it has been argued that single-gene knockout models are insufficient to effectively illustrate the function of these receptors. This demands the use of double or triple knockout models (Tan et al., 2017). So far, the association between GPR41 and kidney injury has not been verified, and extensive studies are needed to investigate and further unravel the relationship between GPCRs and DKD.

### Butyrate treats DKD by triggering autophagy

Recently, Cai et al. (2022) explored the protective effects and mechanism of sodium butyrate on STZ-induced DKD rats through activation of autophagy. They reported that oral sodium butyrate improved blood glucose, serum nitrogen levels, fibronectin, and collagen IV expression. They also confirmed that the beneficial effects of sodium butyrate on the said DKD phenotype were due to the activation of the AMPK/mTOR signalling pathway, which induces autophagy.

Studies by Tang et al. (2022) also showed that sodium butyrate lowered serum creatinine, blood urea nitrogen, and UACR levels in DKD models replicated in db/db mice. They further evaluated the protective effects of butyrate against DKD-induced muscle atrophy. Butyrate provides a protective effect in db/db mice and HG/LPS-induced C2C12 myoblasts by suppressing autophagy and oxidative stress and activating the PI3K/AKT/mTOR pathway. Since the relationship between autophagy and DKD is not entirely deciphered yet, butyrate’s role in the regulation of autophagy and the prevention and treatment of DKD needs further evaluation.

### Butyrate alleviates DKD by novel epigenetic mechanisms

We previously described the epigenetic control by HDAC inhibition mediated by butyrate that leads to amelioration of DKD, which recent studies have corroborated. Du et al. (2020b) found that butyrate alleviated renal dysfunction, serum creatinine and UACR levels, and mesangial matrix expansion in db/db mice by modulating the miR-7a-5p/P311/TGF-β1 pathway. However, no apparent differences in blood glucose levels and body weight were noted. As reviewed by Yang et al. (2021), sodium butyrate significantly improved body weight, urinary microalbumin, and urinary creatinine in diabetic mice by altering lncRNA expression, with no significant improvement in blood glucose levels.

Studies have found that butyrylation, similar to histone acetylation, can competitively inhibit gene transcription (Nie et al., 2017). Also, exogenous crotonate-mediated histone crotonylation inhibits the transcriptional activation of renal inflammatory genes and improves renal function in mice with acute AKI models (Ruiz-Andres et al., 2016). There is also evidence that histone β-hydroxybutyrylation is potentially beneficial for DKD (Luo et al., 2020). However, whether butyrate or sodium butyrate improves DKD renal injury through the histone butyrylation pathway has not been deciphered. Therefore, exploring the role of novel epigenetic mechanisms in DKD gene transcription regulation can provide great insights into the development of novel therapeutics against this disease.

## Conclusion

All in all, endogenous butyrate or exogenous sodium butyrate supplementation improves body weight, glucose, and lipid metabolism, benefits a large population of type 2 diabetes, and protects against DKD, suggesting a new therapeutic reagent for DKD (Hong et al., 2016; Bridgeman et al., 2020). However, *in vivo* experiments have not clarified whether butyrate’s protective effect on DKD is independent or dependent on improving glycolipid metabolism, and the *in vitro* studies have reported that butyrate within a certain concentration range improves HG-induced renal intrinsic cells injury (Huang et al., 2017; Gu et al., 2019), but the harmful effects of butyrate have also been reported when the intervention concentration or time exceeds a certain level (Bock et al., 2013), furthermore, it is unclear whether the beneficial results observed in animal studies can be extrapolated to humans.

**Figure 2:**
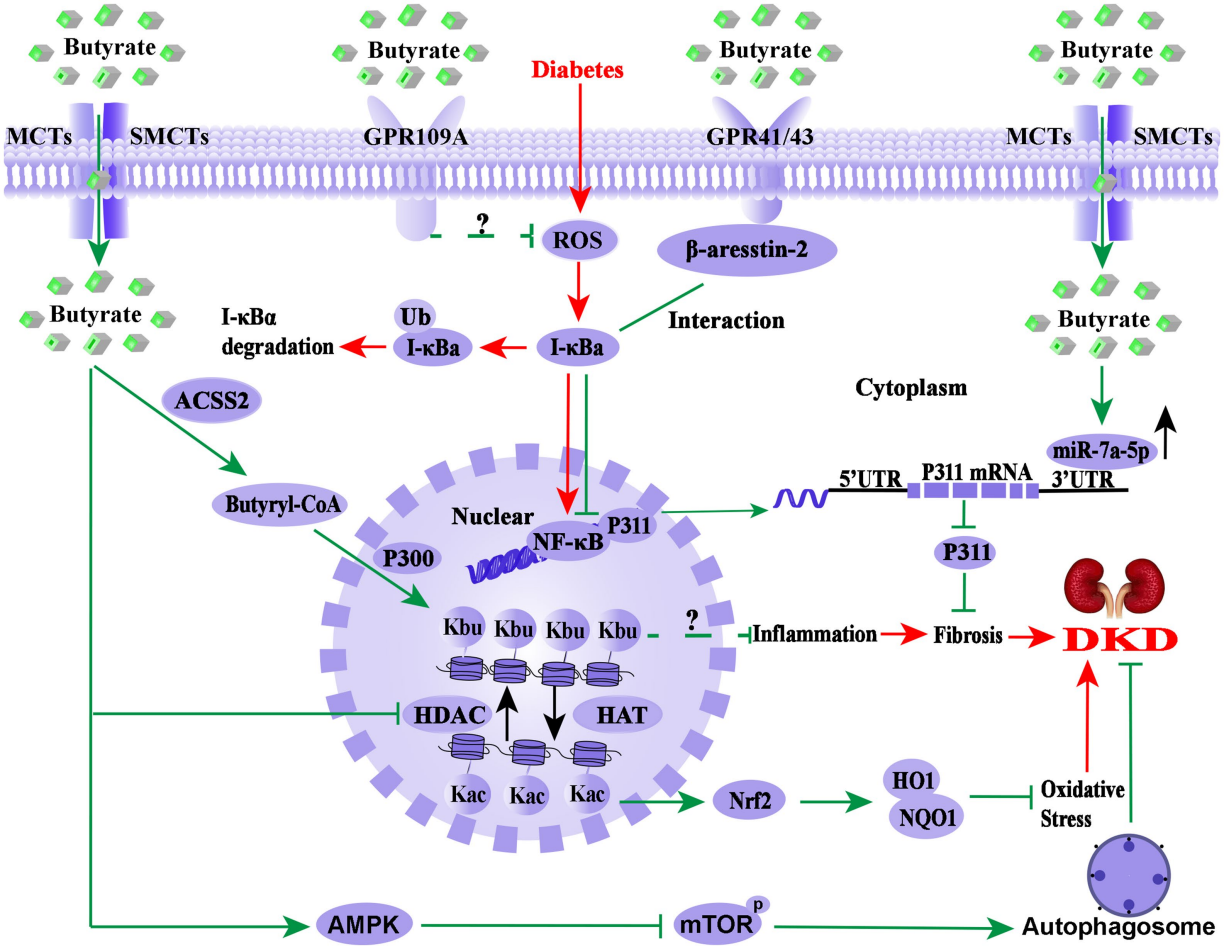
Overview of the molecular mechanism of butyrate in the prevention and treatment of DKD. The pathological process of DKD involves persistent HG-induced oxidative stress, immune system disorders, and inflammation (red arrows). Endogenous or exogenous butyrate (green arrows) inhibits the activity of HDAC, opens the structure of chromatin, and facilitates the expression of the Nrf2 gene, which may enter the nucleus and upregulate the downstream targets HO1 and NQO1 and then inhibits oxidative stress and inflammation in DKD. Meanwhile, GPR43 and GPR109A are important receptors of butyrate for renal protection, and the interaction between β-arrestin-2 and I-κBα is induced by butyrate *via* GPR43, suggesting that butyrate-mediated GPR43-β-arrestin-2 signaling may be a novel and promising target for DKD (green arrows). Moreover, it has been found that butyrate reverses HG-induced the downregulation of miR-7a-5p and inhibits the expression of P311, followed by the inhabitation of the kidney fibrosis of DKD (green arrows) and activated autophagy *via* the AMPK/mTOR pathway to delay the DKD progression. Notably, butyl-CoA, a metabolite of butyrate, is the substrate of histone butyrylation modification, irrespective of whether butyrate or sodium butyrate improves DKD renal injury through histone butyrylation pathway or the cross-talk of the histone post-translational modifications has not been reported. Nrf2, Nuclear factor erythroid 2-related factor 2; HO1, heme oxygenase 1; NQO1, NAD(P)H dehydrogenase quinone 1; HDAC, histone deacetylase; HAT, histone acetyltransferase; UTR, untranslated region; NF-κB, nuclear factor kappa B; Kbu, histone lysine butyrylation; Kac, histone lysine acetylation; ACSS2, acetyl-CoA synthetase 2; p300, a histone acetylation transferase that mediates butyrylation; P311, an RNA-binding protein, which could stimulate fibrosis; AMPK, AMP-activated protein kinase; mTOR, mammalian target of rapamycin.

As shown in Figure 2, the molecular mechanism of butyrate improving DKD is extremely complex. As GPR41, GPR43, or GPR109A agonist, butyrate may exert anti-inflammatory effect through these receptors signalling pathways. At the same time, butyrate also acts as epigenetic regulators in response to the environment or therapeutic modulation by inhibiting HDAC, up-regulation of miR-7a-5p, or induction of the histone butyrylation and autophagy processes. It is worth mentioning that butyrate-induced histone butyrylation, as a novel histone post-translational modification, has demonstrated its renal protective effects on diverse nephropathies, which provides a novel perspective for elucidating the pharmacological mechanisms of butyrate. Therefore, further basic experiments and well-designed clinical studies are necessary to explore the pharmacological effects and molecular mechanism of butyrate in DKD prevention and treatment.

## Author contributions

WH contributed to the conception and design of the research. XC and TZ contributed to the design of the research. YoX contributed to the acquisition and analysis of the data. YuX and YH contributed to the analysis of the data. WH contributed to the acquisition, analysis, and interpretation of the data. All authors drafted the manuscript, critically revised the manuscript, agree to be fully accountable for ensuring the integrity and accuracy of the work, and read and approved the final manuscript. All authors contributed to the article and approved the submitted version.

## Funding

This work was supported by grants from the National Natural Science Foundation of China (Nos. 81800741 and 82170834), the Program of Sichuan Provincial Science and Technology Department (No. 2019YJ0697 and 2018JY0059), and Office of Science Technology and Talent Work of Luzhou (No. 2020LZXNYDP02 and 2021LZXNYD-G01).

## Conflict of interest

The authors declare that the research was conducted in the absence of any commercial or financial relationships that could be construed as a potential conflict of interest.

## Publisher’s note

All claims expressed in this article are solely those of the authors and do not necessarily represent those of their affiliated organizations, or those of the publisher, the editors and the reviewers. Any product that may be evaluated in this article, or claim that may be made by its manufacturer, is not guaranteed or endorsed by the publisher.

## Abbreviations

DKD: Diabetic kidney disease
SCFAs: Short-chain fatty acids
UACR: The ratio of urinary albumin to creatinine
LPS: Lipopolysaccharide
AMPK: AMP-activated protein kinase
β-arrs: β-arrestins
CKD: Chronic kidney disease
STZ: Streptozotocin
GPCRs: G-protein coupled receptors
CoA: Coenzyme A
NF-κB: Nuclear factor-kappa B
mRNA: Messenger RNA
Nrf2: Nuclear factor erythroid 2-related factor 2
mTOR: Mammalian target of rapamycin
miRNA: microRNAs
lncRNAs: Long non-coding RNAs
HDAC: Histone deacetylase
HG: High glucose
